# Major immunophenotypic abnormalities in patients with primary adrenal insufficiency of different etiology

**DOI:** 10.3389/fimmu.2023.1275828

**Published:** 2023-11-15

**Authors:** Hanna F. Nowotny, Thomas Marchant Seiter, Jing Ju, Adrian Gottschlich, Holger Schneider, Stephanie Zopp, Frederick Vogel, Lea Tschaidse, Matthias K. Auer, Christian Lottspeich, Sebastian Kobold, Simon Rothenfusser, Felix Beuschlein, Martin Reincke, Leah Braun, Nicole Reisch

**Affiliations:** ^1^ Department of Medicine IV, LMU University Hospital, LMU Munich, Munich, Germany; ^2^ Division of Clinical Pharmacology, University Hospital, LMU Munich, Munich, Germany; ^3^ Department of Medicine III, LMU University Hospital, LMU Munich, Munich, Germany; ^4^ German Cancer Consortium (DKTK), Partner Site Munich, Munich, Germany; ^5^ Einheit für Klinische Pharmakologie (EKLiP), Helmholtz Munich, Research Center for Environmental Health (HMGU), Neuherberg, Germany; ^6^ Klinik für Endokrinologie, Diabetologie und Klinische Ernährung, Universitätsspital Zürich (USZ) und Universität Zürich (UZH), Zurich, Switzerland

**Keywords:** primary adrenal insufficiency, congenital adrenal hyperplasia, bilateral adrenalectomy, Addison’s disease, immunophenotype, ACTH, cortisol

## Abstract

**Introduction:**

Patients with primary adrenal insufficiency (PAI) suffer from increased risk of infection, adrenal crises and have a higher mortality rate. Such dismal outcomes have been inferred to immune cell dysregulation because of unphysiological cortisol replacement. As the immune landscape of patients with different types of PAI has not been systematically explored, we set out to immunophenotype PAI patients with different causes of glucocorticoid (GC) deficiency.

**Methods:**

This cross-sectional single center study includes 28 patients with congenital adrenal hyperplasia (CAH), 27 after bilateral adrenalectomy due to Cushing’s syndrome (BADx), 21 with Addison’s disease (AD) and 52 healthy controls. All patients with PAI were on a stable GC replacement regimen with a median dose of 25 mg hydrocortisone per day. Peripheral blood mononuclear cells were isolated from heparinized blood samples. Immune cell subsets were analyzed using multicolor flow cytometry after four-hour stimulation with phorbol myristate acetate and ionomycin. Natural killer (NK-) cell cytotoxicity and clock gene expression were investigated.

**Results:**

The percentage of T helper cell subsets was downregulated in AD patients (Th1 p = 0.0024, Th2 p = 0.0157, Th17 p < 0.0001) compared to controls. Cytotoxic T cell subsets were reduced in AD (Tc1 p = 0.0075, Tc2 p = 0.0154) and CAH patients (Tc1 p = 0.0055, Tc2 p = 0.0012) compared to controls. NKCC was reduced in all subsets of PAI patients, with smallest changes in CAH. Degranulation marker CD107a expression was upregulated in BADx and AD, not in CAH patients compared to controls (BADx p < 0.0001; AD p = 0.0002). In contrast to NK cell activating receptors, NK cell inhibiting receptor CD94 was upregulated in BADx and AD, but not in CAH patients (p < 0.0001). Although modulation in clock gene expression could be confirmed in our patient subgroups, major interindividual-intergroup dissimilarities were not detected.

**Discussion:**

In patients with different etiologies of PAI, distinct differences in T and NK cell-phenotypes became apparent despite the use of same GC preparation and dose. Our results highlight unsuspected differences in immune cell composition and function in PAI patients of different causes and suggest disease-specific alterations that might necessitate disease-specific treatment.

## Introduction

This study focuses on three different subtypes of primary adrenal insufficiency (PAI) with either an inborn lack of cortisol but maintained adrenal androgen synthesis in congenital adrenal hyperplasia (CAH) versus complete loss of adrenal function over time due to an autoimmune disorder in Addison’s disease (AD) or abruptly due to bilateral adrenalectomy (1, 2). BADx is recommended as a second-line therapy for occult or metastatic ectopic adrenocorticotropic hormone (ACTH) secretion, as a life-preserving emergency treatment or ultima ratio in patients with severe treatment-refractory ACTH-dependent Cushing’s syndrome (CS) and in the rare event of endogenous adrenal CS due to bilateral disease (3–5).

Adrenal insufficiency (AI) requires life-long glucocorticoid (GC) substitution therapy as well as stress adaptation in order to prevent life-threatening events of adrenal crises. This is usually accomplished by replacing hydrocortisone (HC) with a total daily dose of 15-25 mg divided into two to three doses with the highest in the morning to mimic the circadian rhythmicity of cortisol secretion (6–9).

The physiologic rise in serum cortisol levels as a reaction to stressful events such as systemic infection with fever can only be achieved by patients with AI by temporarily elevating their required GC dose (10). Acute deficiency of cortisol results in adrenal crisis, triggering a rise in cytokine levels and altered immune cell populations due to the missing physiological suppressive function of cortisol. This contributes to a systemic response including hypotension, hyponatremia, hyperkalemia and severe fatigue and weakness (11, 12).

Episodes of adrenal crises are common in patients with PAI ranging from 4.9-5.8/100 patient years (py) (13–15) in CAH to 8.3 and 9.3 events per 100 py in patients with AD or following BADx, respectively. This goes in hand with a higher mortality rate in PAI patients compared to healthy controls in population-based studies with increased risk ratios of 2-3 in AD (16) as well as increased hazard ratios of 2-3 in BADx (17) and CAH (18).

To unravel underlying immunological causes of the increased risk of adrenal crisis in these patients, Bancos et al. already investigated natural killer cell (NK cell) function in a cross-sectional study of 42 PAI patients. They could demonstrate an inhibition of NK cell cytotoxicity (NKCC) by means of decreased expression of NK cell activating receptors, which was linked to increased incidence of respiratory infections in this patient cohort (19). Further prospective trials demonstrated a pro-inflammatory state in PAI patients with dysregulations in immune cell profiles, such as increased numbers of classical monocytes and decreased numbers of CD16^+^ NK cells (20). As immune cell profiles could be restored after switch from standard GC substitution therapy to modified-release HC preparation – which more accurately mimics physiologic circadian rhythmicity of cortisol secretion – it was proposed that modulations of clock gene expression would explain these changes, which could ultimately be shown in an ancillary study (20, 21).

However, to the best of our knowledge there are no studies explicitly analyzing the specific immunophenotype in PAI patients with different causes of GC deficiency compared to healthy controls. We hypothesized that due to the nature and pathophysiology of these different causes of PAI, they might ultimately have different impact on immunophenotype beyond the sole effect of GC replacement therapy.

## Methods

### Subjects

In total 128 patients were recruited from the endocrine outpatient clinic of the University Hospital Munich, Germany, including 28 patients with classic CAH, 27 patients after BADx due to CS (Cushing’s disease (n=17), adrenal (n=7) and ectopic CS (n=3)), 21 patients with isolated AD (AD (n=4), autoimmune polyglandular syndrome type 2 (n=15)) and 52 age-, sex- and BMI-matched healthy controls. Of those patients with autoimmune polyglandular syndrome type 2, two patients suffered from diabetes mellitus type I and one patient from autoimmune gastritis. Therefore, the majority of patients with AD had Hashimoto’s thyroiditis (15 patients with APS2) and also some of the patients in the other cohorts suffered from Hashimoto’s thyroiditis with euthyreosis on L-thyroxin replacement (3 BADx, 4 CAH, 1 control). As exclusion of these patients in the subgroup of BADx, CAH and controls did not alter the results, they were not excluded from the analysis. All patients and controls provided written informed consent to participate in the German Cushing’s Registry (NeoExNET, ethical approval no. 152-10) or our registry and biobank for adrenal insufficiency and differences of sex development (Bio AI/DSD, ethical approval no. 19-558). Hypercortisolism was excluded in all control patients by use of recommended standard biochemical testing including low dose dexamethasone suppression test, late-night salivary cortisol and urinary free cortisol in a 24-h collection using validated assays. Adrenal insufficiency was excluded upon clinical suspicion. Exclusion criteria for healthy controls were intake of GCs in the last 12 months, current/past malignancy, autoimmune diseases other than Hashimoto’s thyroiditis or immunosuppressive/immunomodulatory medication. Patient samples were recruited during a regularly conducted follow-up visit between 8-12 am after intake of morning medication.

### Isolation of immune cells

Blood samples were collected over all four seasons in all of the analyzed patient subgroups. Heparinized blood samples were processed for the isolation of peripheral blood mononuclear cells (PBMC) on the same day (withing 12 hours) to minimize apoptotic effects. We collected three tubes (each 7.5 ml) of heparinized blood from each patient to isolate PBMCs using a standardized protocol for density centrifugation. All isolated cells were cryopreserved using 10% dimethyl sulfoxide (DMSO, Sigma-Aldrich^®^) in fetal calf serum (FCS, Thermo Fisher Scientific®). Cells were cryopreserved at -80°C using an isopropanol freezing chamber (Mr. Frosty®, Sigma-Aldrich®) before further analysis by multicolor flow cytometry.

### Thawing and staining of PBMCs

Cryopreserved PBMCs were first thawed in a 37°C water bath and washed in complete medium (450 x g, RT, 10 minutes) before incubating at 37°C and 5% CO_2_ for overnight rest. Before staining, samples were stimulated using working concentrations of phorbol myristate acetate (PMA)/ionomycin (eBioscienceTM Cell Stimulation Cocktail (500X), diluted 1/500 with 2µl/ml final concentration, InvitrogenTM). PMA mimics the function of diacylglycerol by diffusing into the cytoplasm and activating Protein Kinase C (PKC). In combinatory use with ionomycin, it results in a moderate level of cytokine release (22). After blocking of unspecific binding sites with Human TruStain FcX®, BioLegend®, cells were stained with appropriate anti-human antibodies or their correspondent isotype control (panels shown below) for 30 minutes at 4°C with a concentration of 0.7/100 µl.

For further intracellular staining required for the T cell panel we used the eBioscienceTM Foxp3/Transcription Factor Staining Buffer Set (Invitrogen®) according to the manufacturer’s protocol again using PMA/ionomycin as stimulating agents and Brefeldin A (Invitrogen®) as a protein transport inhibitor.

Samples were fixed using 4% paraformaldehyde (PFA), before transfer to Dulbecco’s Balanced Salt Solution (DPBS) for analysis by multicolor flow cytometry.

### Assessment of T cell and NK cell subsets and surface phenotypes

We performed analysis of immune cell subsets via multicolor flow cytometry for T cell subsets under unstimulated and stimulated conditions, respectively. The T cell panel consisted of CD3-PECy5 (Clone HIT3a), CD4-BV786 (Clone OKT4), CD8-PECy7 (Clone RPA-T8), CD25-BV510 (Clone M-A251), FOXP3-BV421 (Clone 206D), IFN-γ-BV605 (Clone 4S.B3), IL-4-AF488 (MP4-25D2), IL-9-PE (Clone MH9A4), IL-17A-BV711 (Clone BL168) and IL-22-APC (Clone 2G12A41). The NK cell panel included the following antibodies: CD45-BV605 (Clone HI30), CD3-PECy5 (Clone HIT3a), CD56-FITC (Clone HCD56), CD16-BV785 (Clone 3G8), NKG2D-BV510 (Clone 1D11), NKp30-PE (Clone P30-15), NKp46-BV711 (Clone 9E2), CD107a-BV421 (Clone H4A3), NKG2A-PECy7 (Clone S19004C), CD94-PerCPCy5.5 (Clone DX22) and KIR-APC (Clone Clone HP-MA4).

All antibodies and their respective isotype controls were purchased from BioLegend®. All cytometric measurements were performed using LSFortessa II® (BD Biosciences®) and FlowJo® 10.8.1 software.

In the following, data is presented in terms of percentage of parent population.

### Assessment of Natural Killer cell cytotoxicity

NKCC was evaluated *in vitro* by co-cultivating isolated PBMCs (effector-cells, E) with K562 cells (targeT cells, T) for 4 hours at 37°C and 5% CO_2_ in three replicates. Two E:T-ratios of 50:1 and 200:1 were used. K562 cells were purchased from ATCC (USA). Cells were lentivirally transduced with a pCDH-EF1a-eFly-eGFP plasmid (23) and enhanced green-fluorescent protein (eGFP) positive cells were sorted with a BD FACSAria™ III Cell Sorter. STR profiling was carried out at the Institute for forensic medicine of the LMU Munich to ensure correct origin of all cell lines. Mycoplasma contamination was excluded by polymerase chain reaction (PCR). Cells were cultured in RPMI containing 20% FBS, 2 mM L-Glutamine, 100 U/ml penicillin and 100 µg/ml streptomycin. All cells were grown at 37°C in a humidified incubator with 5% CO_2_. Cells were stored at -80°C or in the liquid nitrogen after addition of freezing medium (90% FCS and 10% DMSO).

The assay was performed using the Bio-GloTM Luciferase assay system (Promega®) according to the manufacturer’s protocol. TargeT cell killing is presented as specific lysis in 1 - (Luminescence of sample/negative control) x 100%.

### Analysis of clock gene expression

After ribonucleic acid (RNA)-isolation using the Quick-RNA™ Miniprep Kit (Zymo Research®) and complementary deoxyribonucleic acid (cDNA) transcription, qPCR analysis was used to quantify mRNA expression of the following clock genes: circadian locomotor output cycles kaput *(CLOCK)*, aryl hydrocarbon receptor nuclear translocator-like protein 1 *(ARNTL)*, nuclear receptor subfamily 1 group D member 1 *(NR1D1)*, Cryptochrome Circadian Regulator 1 *(Cry1)* and *Cry2*, WEE1 G2 Checkpoint Kinase *(WEE1)*, Timeless Circadian Regulator *(TIMELESS)*, CAMP Responsive Element Binding Protein 1 *(CREB1)* and Period Circadian Regulator 3 *(PER3)*. Data was obtained as cycle threshold (Ct), and ΔCt = (Ct of target gene) - (Ct of *β-Actin*). Relative mRNA expression was expressed according to the delta delta CT method calculated by the formula 2^-ΔΔCt^. ΔΔCt = (ΔCt of patients) - (ΔCt of mean of controls). Some samples could not be analyzed due to missing sample volume [controls (n=32), CAH (n=24), AD (n=13), BADx (n=19)].

### Statistical analysis

Based on a *post-hoc* analysis of previously analyzed data, with an effect size of d = 0.858 and a power of (1-β) = 0.947, we calculated that a total sample size of 30 patients is required at an α-error of 0.10 (adjusted α-error for rare diseases). For statistical analysis data was tested for normality using the Shapiro-Wilk test. Column statistics were calculated using GraphPad Prism (Mean, SEM, Quartiles). Non-normally distributed data were analyzed using Kruskal-Wallis test and Dunn’s multiple comparisons test. The confidence interval was defined as 95% and a p-value of < 0.05 was considered statistically significant P ≤ 0.05 (*), ≤ 0.01 (**), ≤ 0.001 (***), < 0.0001 (****)). Statistical analysis and graphical presentation were carried out using GraphPad Prism 7.03 and Adobe Illustrator 2020.

## Results

### Characteristics of study participants

As summarized in **Table 1**, 128 patients were recruited in our outpatient clinic including 28 patients with classic CAH due to 21-hydroxylase deficiency (71.4% females, 28.6% males), 27 after BADx (77.8% females, 22.2% males) and 21 patients with AD (66.7% females, 33.3% males) as well as 52 healthy controls (76.9% females, 23.1% males). Patients with AD [median 58 years (interquartile range (IQR) 17)] or after BADx [median 55 years (IQR 28)] did not significantly differ from controls [median 51 (IQR 29.5) years] regarding age, however patients with CAH [median 29 years (IQR 24.8)] were younger. Therefore, the CAH subgroup was split into patients above and below the age of 30 resulting in a subgroup of patients with median age of 50 years comparable to the other patient groups. As there were no differences between younger and older CAH patients regarding the results on immunophenotype data is not shown in the manuscript.

**Table 1:** Patient characteristics.

	CAH (1)	BADx (2)	AD (3)	Controls (4)	p
	n = 28	n = 27	n = 21	n = 52	
sex					0.7750
female	20 (71.4)	21 (77.8)	14 (66.7)	40 (76.9)	
male	8 (28.6)	6 (22.2)	7 (33.3)	12 (23.1)	
age (years)	29 (24.8)	55 (28)	58 (17)	51 (29.5)	<0.0001^(1vs.2/3/4)^
BMI (kg/m^2^)	24.51 (4.9)	26.8 (6.9)	25.08 (4.6)	24.90 (8.19)	0.3097
HC dose (mg)	25 (10)	25 (5)	25 (5)	n.a.	0.5504
HC/BMI (mg/(kg/m^2^))	0.98 (0.50)	0.88 (0.28)	1.12 (0.40)	n.a.	0.2523
HC/BSA (mg/m^2^)	15.85 (4.5)	13.22 (4.3)	13.01 (4.6)	n.a.	0.2005
Fludrocortisone treatment	17 (60.7)	26 (96.3)	16 (66.7)	n.a.	0.0070^(1vs.2)^
Fludrocortione dose (mg)	0.05 (0.1)	0.1 (0.05)	0.075 (0.1)	n.a.	0.0221^(1vs.2)^

CAH, congenital adrenal hyperplasia; BADx, bilateral adrenalectomy; AD, Addison’s disease; BMI, Body-Mass-Index; HC, Hydrocortisone; BSA, Body surface area. P-value refers to group differences of Kruskal-Wallis test. Shown are median (IQR). Significant results of Dunn’s multiple comparisons are depicted as superscript numbers where CAH (1), BADx (2), AD (3) and Controls (4). Eg.: 1vs.2/3/4 depicts statistically significant differences in the following pairwise comparisons: “CAH and BADx”, “CAH and. AD” and “CAH and controls”.

BMI did not significantly differ between the patient subgroups (**Table 1**). All patients were of the same ethnicity. All patients with PAI were on a stable GC replacement regimen using twice or thrice daily administration of HC. A median dose of 25 mg was used in all subtypes of PAI with no significant difference in HC/BMI or body surface area (BSA) between the subgroups.

Mineralocorticoid replacement was used in 17 (60.7%) of patients with CAH, 26 (96.3%) of BADx patients and 16 (76.2%) of patients with AD. Besides use of mineralocorticoid treatment, doses used also varied between a median of 0.05 – 0.1 mg/d as seen in **Table 1**.

### Dysregulation of T cell subsets in different types of PAI

Analysis of T cell subsets using multicolor flow cytometry revealed a lower percentage of CD3^+^ T cells in BADx patients [median 53.7% (range 47.5 – 61.0%)] compared to those with CAH [median 61.5% (range 58.0 – 69.1%), p = 0.003] (compare **Table 2**). The percentage of CD4^+^ T cells was generally lower in patients with CAH [median 68.2% (range 60.7 – 75.9%), p = 0.0035] or after BADx [median 63.9% (range 49.0 – 82.7%)] compared to controls. Cytotoxic CD3^+^CD8^+^ T cells (Tc) were upregulated in patients with BADx [median 22.5% (range 13.2 – 41.7%)] compared to healthy controls [median 13.1% (range 7.8 – 16.5%), p = 0.0011].

**Table 2:** Differences in T cell subsets in subgroups of PAI patients compared to healthy controls.

T cells	CAH (1)	BADx (2)	AD (3)	Controls (4)	p-value
**CD3+ T cells**	61.5 (58.0 – 69.1)	53.7 (47.5 – 61.0)	57.3 (51.8 – 62.3)	57.4 (52.5 -63.4)	0.0063^(1vs.2)^
**CD4+ T cells**	68.2 (60.7 – 75.9)	63.9 (49.0 – 82.7)	79.4 (73.5 – 84.5)	77.1 (71.2 – 83.7)	0.0003^(1/2vs.3/4)^
**IFN-γ+ T_H_-cells (Th1)**	12.9 (7.7 – 19.0)	17.4 (8.9 – 25.9)	8.1 (4.8 – 16.4)	18.0 (10.4 – 28.0)	0.0026^(3vs.2/4)^
**IL-4+ T_H_-cells (Th2)**	3.4 (2.4 – 5.3)	5.8 (3.7 – 10.1)	2.4 (1.6 – 3.9)	4.4 (3.0 – 6.2)	0.0011^(3vs.2/4)^
**IL-17+ T_H_-cells (Th17)**	9.6 (5.1 – 15.6)	11.4 (6.1 – 13.7)	1.7 (1.2 – 2.4)	8.7 (11.1 – 13.6)	<0.0001^(3vs.1/2/4)^
**IL-9+ T_H_-cells (Th9)**	2.1 (0.9 – 3.8)	3.3 (1.2 – 6.5)	0.5 (0.2 – 0.7)	0.2 (0.2 – 0.6)	<0.0001^(1/2vs.3/4)^
**IL-22+ T_H_-cells (Th22)**	0.6 (0.4 – 1.1)	2.9 (0.9 – 5.4)	0.4 (0.2 – 1.0)	0.8 (0.5 – 1.2)	<0.0001^(1vs.2/4,2vs.4)^
**CD8+ T cells**	17.6 (12.4 – 23.4)	22.5 (13.2 – 41.7)	14.7 (9.7 – 19.0)	7.8 (13.1 – 16.5)	0.0009^(2vs.4)^
**IFN-γ+ T_C_-cells (Tc1)**	39.2 (25.7 – 49.9)	58.8 (30.2 – 66.1)	39.0 (26.3 – 46.5)	36.7 (56.2 – 69.2)	0.0004^(4vs.1/3)^
**IL-4+ T_C_-cells (Tc2)**	4.2 (3.0 – 7.0)	14.5 (5.4 – 23.5)	2.5 (1.2 – 5.9)	6.8 (3.9 – 10.8)	<0.0001^(2vs.1/3,3vs.4)^
**IL-17+ T_C_-cells (Tc17)**	12.6 (6.1 – 16.3)	12.9 (8.4 – 18.2)	1.9 (1.1 – 3.6)	16.6 (13.5 – 18.9)	<0.0001^(1vs.3/4,2vs.3,3vs.4)^
**IL-9+ T_C_-cells (Tc9)**	7.3 (4.1 – 15.6)	13.2 (6.4 – 18.6)	3.4 (2.4 – 7.9)	4.2 (2.2 -8.9)	<0.0001^(2vs.3/4)^
**IL-22+ T_C_-cells (Tc22)**	0.3 (0.1 – 0.9)	1.4 (0.6 – 5.3)	0.2 (0.04 – 1.0)	0.5 (0.3 – 0.8)	<0.0001^(2vs.1/3/4)^

P-value refers to group differences of Kruskal-Wallis test. Significant results of Dunn’s multiple comparisons are depicted as superscript numbers where CAH (1), BADx (2), AD (3) and Controls (4). Eg.: 1vs.2/3/4 depicts statistically significant differences in the following pairwise comparisons: “CAH and BADx”, “CAH and. AD” and “CAH and controls”.

**Figure 1:**
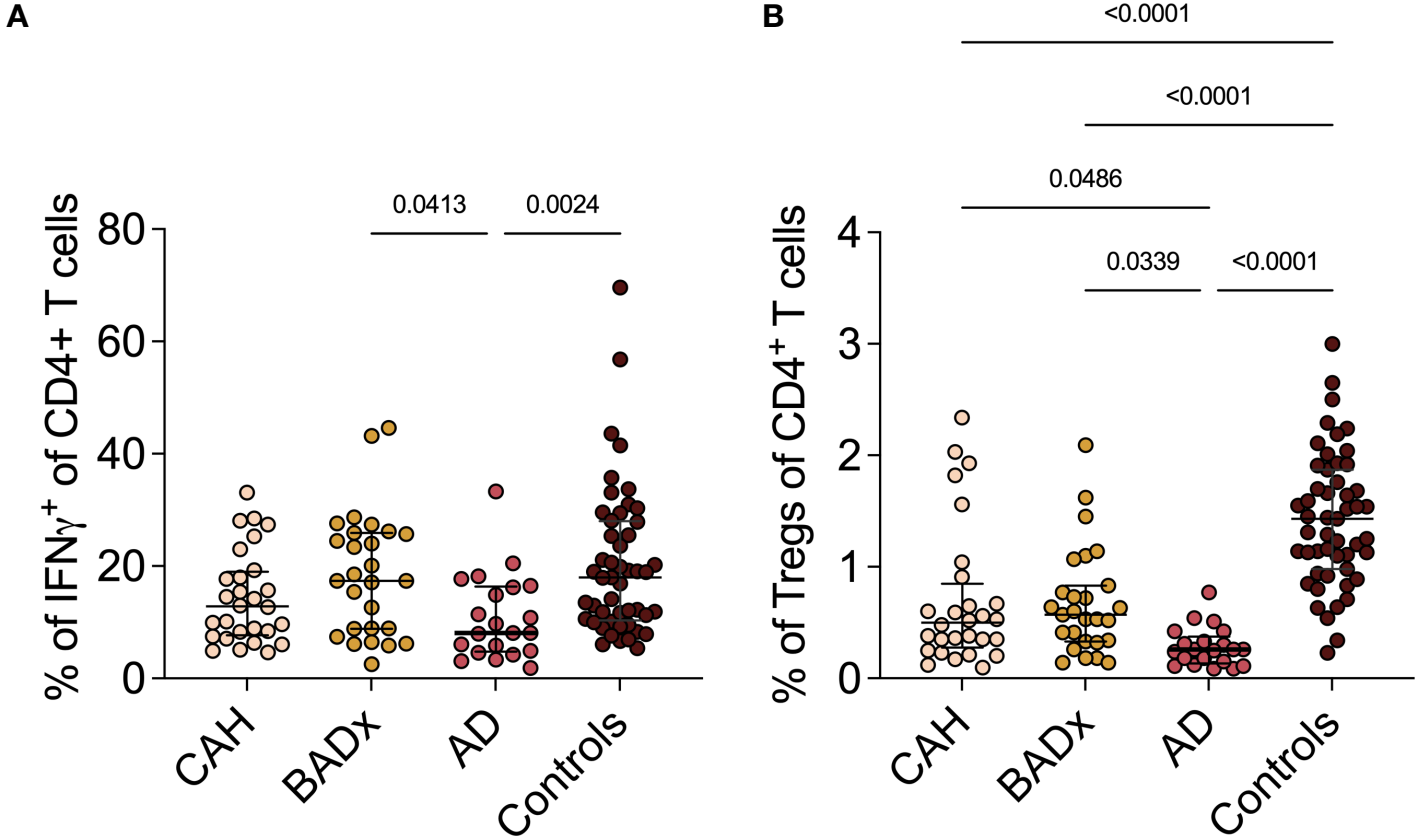
Dysregulation of Th1 and Treg subsets in PAI patients. Whole PBMCs were analyzed by multicolor flow cytometry for expression of different T cell subset specific cytokines. Percentage of **(A)** IFNγ^+^ Th1 cells and **(B)** CD3^+^CD4^+^CD25^+^FoxP3^+^ Tregs is presented in terms of percentage of parent population. Samples were analyzed after 4 hour stimulation with PMA/ionomycin. Kruskal–Wallis test and Dunn’s multiple comparisons test were used for statistical analysis. P-values are indicated in the pairwise comparison. A p-value of < 0.05 was considered statistically significant.

Interferon-gamma (IFNγ) producing Th1 cells were reduced in AD patients (median 8.1% (range 4.8 – 16.4%) compared to controls (median 18.0% (range 10.4 – 28.0, p = 0.0024) as shown in **Table 2** and **Figure 1A**. The same effect was seen in Tc1 cells (AD median 39.0% (range 26.3 – 46.5) vs. controls median 56.2% (range 36.7 – 69.2%), p = 0.0075), with however similar effects in CAH patients [median 39.2 (range 25.7 – 49.9), p = 0.0055].

In the same way, the strongest reduction in Interleukin-4 (IL-4) secreting Th2 or Tc2 cells was observed in AD patients (Th1 median 2.4% (range 1.6 – 3.9%), p = 0.0157; Tc1 median 2.5% (range 1.2 – 5.9%), p = 0.0075) compared to controls (median 4.4% (range 3.0 – 6.2%); Tc1 median 6.8% (range 3.9 – 10.8%)).

The same effect could be observed regarding Interleukin-17 (IL-17) secreting Th17 and Tc17 cells. The percentage of Th17 and Tc17 cells was most reduced in the subgroup of AD patients [Th17 median 1.7% (range 1.2 – 2.4%), p < 0.0001; Tc17 median 1.9% (1.1 – 3.6%), p < 0.0001] compared to controls [Th17 median 8.7% (11.1 – 13.6%); Tc17 median 16.6% (range 13.5 – 18.9%)].

Interleukin-9 (IL-9) secreting Th9 cells were upregulated in both CAH [median 2.1% (range 0.9 – 3.8%), p < 0.0001] and BADx patients [median 3.3% (range 1.2 – 6.5%), p < 0.0001] compared to controls [median 0.2% (range 0.2 – 0.6%)] with no major difference in AD patients [median 0.5% (range 0.2 – 0.7%), p > 0.9999].

Similarly, percentage of Interleukin-22 (IL-22) positive Th22 and Tc22 cells was higher in BADx patients compared to a healthy control cohort [Th22 BADx median 2.9% (range 0.9 – 5.4%) vs. controls median 0.8% (range 0.5 – 1.2%), p = 0.0009; Tc22 BADx median 1.4% (range 0.6 – 5.3%) vs. controls median 0.5% (range 0.3 – 0.8%), p = 0.0149].

Regarding the subset of CD4^+^CD25^+^FoxP3^+^ regulatory T cells (Tregs), the percentage of Tregs (% of CD25^+^Foxp3^+^ cells of all CD4 ^+^ T cells) was reduced in CAH (median 0.50% (range 0.28 – 0.85%), p < 0.0001), BADx [median 0.57% (range 0.33 – 0.83), p < 0.0001] and AD patients [median 0.26% (range 0.14 – 0.38), p < 0.0001] compared to controls (median 1.43% (range 0.98- 1.87%), with the lowest percentage of Tregs present in the subgroup of AD patients (CAH vs. AD p = 0.0486 and BADx vs. AD p = 0.0339, **Figure 1B**).

As use of mineralocorticoid treatment and dose of fludrocortisone replacement differed between the different PAI subtypes, we also analyzed the effect on T cell subsets by correlation analysis. The presence of mineralocorticoid treatment correlated with the percentage of stimulated Th22 cells (r = 0.3239, p = 0.0043) and the dose of fludrocortisone with the percentage of stimulated Th1 cells (r = 0.2929, p = 0.0043). There was however no statistically significant correlation between HC dose and levels of T cell subsets nor between daily HC and mineralocorticoid dose used.

### Dysregulation of NK cell receptor expression and impact on NK cell cytotoxicity in different types of PAI

**Figure 2:**
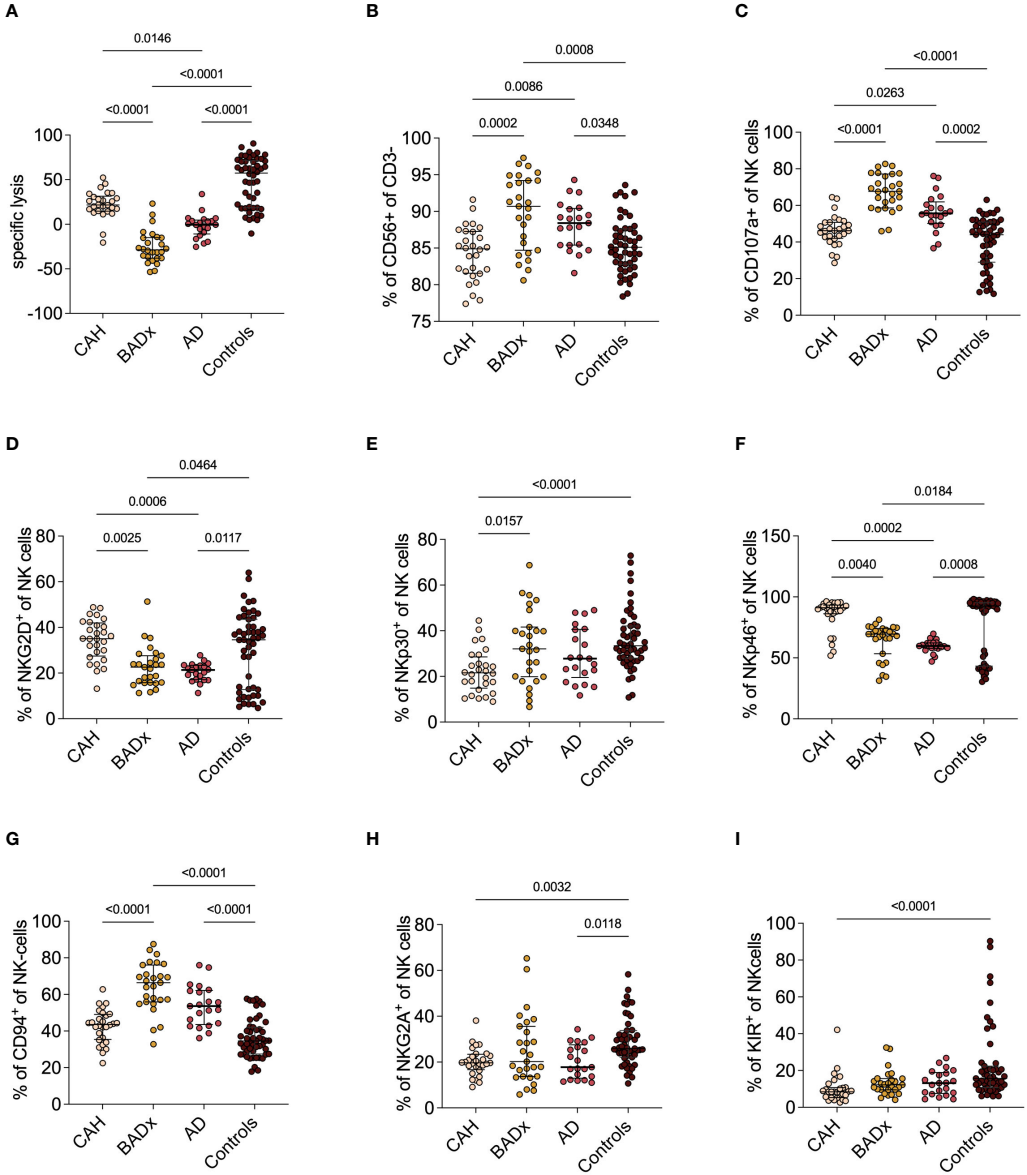
NK cell phenotype and function in different etiologies of PAI. **(A)** NKCC was analyzed using a luciferase-based killing assay including whole PBMCs as effector cells and luciferase and GFP-transduced K562 cells at an E:T of 200:1. Data presented in terms of specific lysis (luminescence of sample/positive control). **(B-I)** Whole PBMC samples were analyzed by multicolor flow cytometry for expression of different NK cell markers, including analysis of whole percentage of **(B)** CD3^-^CD56^+^ NK cells, **(C)** percentage expression of degranulation marker CD107a **(C-F)** percentage expression of activating receptors NKG2D, NKp30 and NKp46 and **(G-I)** percentage expression of inhibiting receptors CD94, NKG2A and KIR. Data is presented in terms of percentage of parent population. Samples were analyzed after 4 hour stimulation with PMA/ionomycin. Kruskal–Wallis test and Dunn’s multiple comparisons test were used for statistical analysis. P-values are indicated in the pairwise comparison. A p-value of < 0.05 was considered statistically significant.

In addition to the above illustrated effects on T cell subsets, we could show that NK cell cytotoxicity is reduced in all subsets of PAI patients using a luciferase-based killing assay (**Figure 2A**). While at an effector to target cell ratio of 200:1 median specific lysis was 57.5% (range 18.6 – 71.3%) in the control cohort, a median specific lysis of 21.7% (14.7 – 31.6%) was detected in the subgroup of CAH patients and even lower rates of specific lysis was observed in AD (-0.5% (range -10.8 – 4.7%)) and for patients after BADx (median -28.7% (range -37.9 – (-14.8)%). Negative values indicate insufficient killing capacity resulting in target cell replication.

At the same time, we could observe an upregulation of the percentage of NK cells compared to controls (BADx median 90.7% (range 84.7 – 94.2%), p = 0.0008; AD median 88.4% (range 85.4 – 90.4%), p = 0.0348; controls median 85.1% (range 82.8 – 87.6%) also expressed as an increase in CD107a expression as a marker of NK cell degranulation (BADx median 67.7% (range 58.6 – 77.2%), p < 0.0001; AD median 55.7% (range 50.2 – 61.9%), p = 0.0002; controls median 44.0% (range 29.0 – 50.9%)) (**Figures 2B, C**). The percentage of NK cell activating receptor expressing NK cells, such as natural killer group 2D (NKG2D), NK cell-protein with 30/46 kilodalton (NKp30 and NKp46) did either not differ (NKp30) or was even slightly lower compared to levels of controls (NKG2D: BADx median 22.6% (range 15.8 – 27.6%), p = 0.0464; AD median 21.4% (range 17.1 – 23.4%) p = 0.0117; controls median 34.6% (range 12.8 – 43.5%); NKp46: BADx median 69.6% (range 53.4 – 74.1%), p = 0.0184; AD median 59.8% (range 57.9 – 62.3%), p = 0.0008; controls median 92.5% (range 43.3 – 95.2%)) (compare **Figures 2D-F**). Percentage expression of NK cell inhibiting receptor CD94, however, was upregulated in both BADx and AD patients [BADx median 66.4% (range 55.9 – 76.1%), p < 0.0001; AD median 53.6% (range 43.6 – 62.2%), p < 0.0001; controls median 34.6% (range 27.5 – 42.0%)] (**Figures 2G-I**).

The use of mineralocorticoid replacement therapy correlated with CD94 expression (r = 0.2289, p = 0.0467), as well as use of mineralocorticoids and fludrocortisone dose with NKp30 expression (r = 0.2562, p = 0.0255; dose: r = 0.2316, p = 0.0441).

### CLOCK Gene expression in patients with different causes of PAI

**Figure 3:**
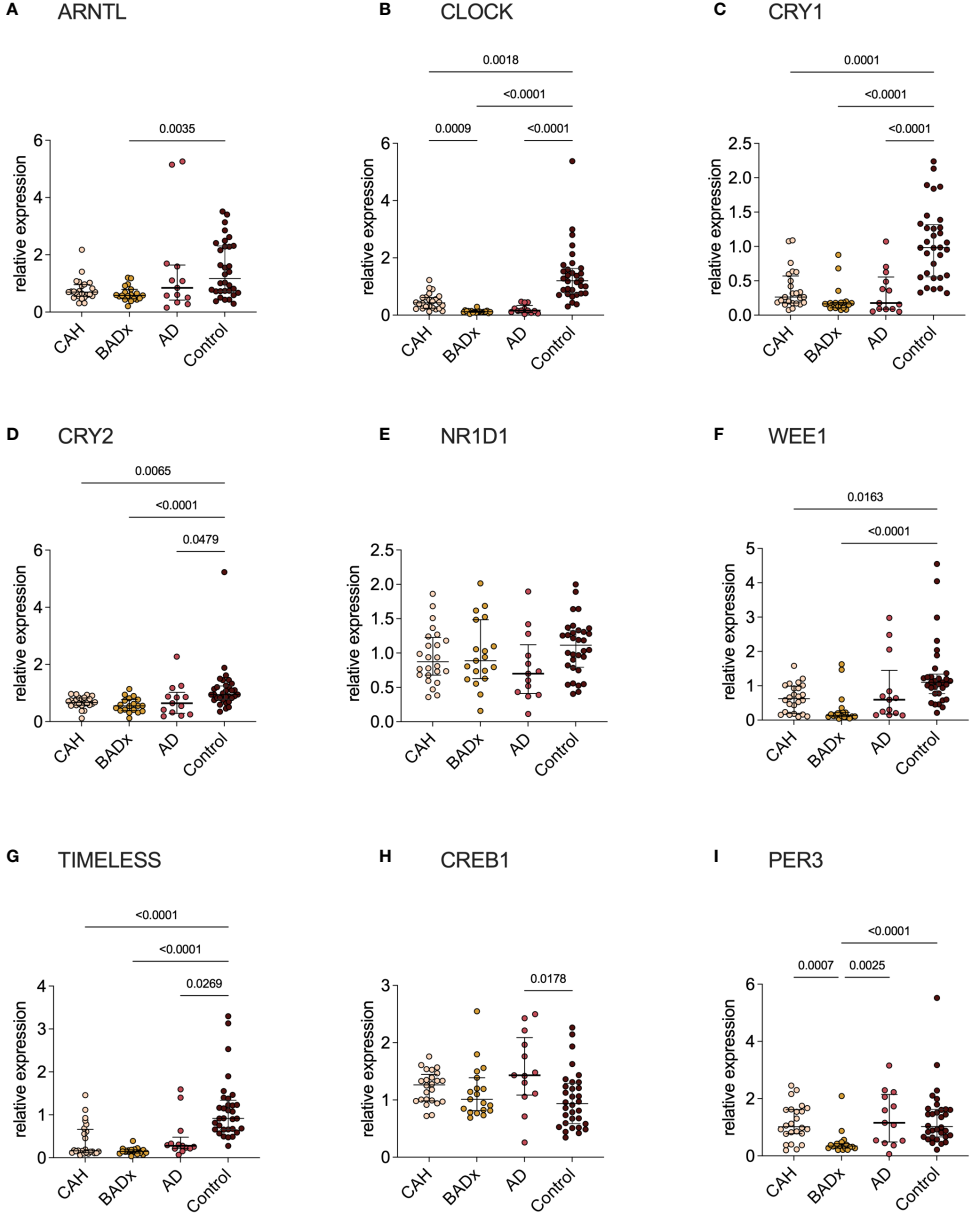
Clock gene expression is equally impaired in all three subtypes of PAI. mRNA expression of different clock genes [**(A)** – ARNTL, **(B)** – CLOCK, **(C)** – CRY1, **(D)** – CRY2, **(E)** – NR1D1, **(F)** – WEE1, **(G)** – TIMELESS, **(H)** – CREB1, **(I)** – PER3] was analyzed using qPCR analysis after RNA isolation of whole PBMCs and cDNA transcription. Data is presented as relative mRNA expression. Kruskal–Wallis test and Dunn’s multiple comparisons test were used for statistical analysis. P-values are indicated in the pairwise comparison. A p-value of < 0.05 was considered statistically significant.

Analysis of relative mRNA expression of different clock genes in corresponding PBMC samples of the same patient subgroups revealed significant differences in *CLOCK*, *CRY1*, *CRY2, WEE1* (no change in AD patients) and *TIMELESS* expression in all subgroups with PAI compared to controls, despite a difference regarding *CLOCK* expression in CAH and BADx patients (**Figure 3**). *ARNTL* expression was downregulated in BADx patients compared to controls (**Figure 3A**, p=0.0035). *CREB1* expression was upregulated in AD patients (**Figure 3H**, p = 0.0178), while *PER3* expression was downregulated in BADx patients compared to all other patient subgroups (**Figure 3I**, p<0.0001). No differences were observed regarding relative *NR1D1* expression (**Figure 3E**, p=0.1257).

## Discussion

The main findings of our study in a cohort of PAI patients due to three different etiologies – inborn defect of steroidogenesis in CAH, autoimmune destruction of adrenocortical cells in AD and surgical removal of the adrenal glands due to CS – are distinct differences in immunophenotype that became apparent despite the use of the same GC preparation and dose in all three groups. This includes relevant changes in levels of Th/Tc1, Th/Tc2, Th/Tc17, Th9, Th/Tc22 and NKCC which might account for higher rates of intercurrent infections and higher incidences of related autoimmune disorders (19, 24–28). Previous publications demonstrated a dysregulation of immune-cell profiles and an impairment of immune function in PAI patients under conventional GC therapy, which was proposed to be the effect of modulations of clock gene expression, as a switch to modified-release preparations restored immunophenotype (19–21). Although modulation in clock gene expression could be confirmed in all our patient subgroups (**Figure 3**), major interindividual-intergroup dissimilarities were not detected.

While pleiotropic effects of both endogenous and exogenous GCs on the immune system have long been described, it appears illusive to summarize these immunosuppressive and immunoregulatory cellular and molecular mechanisms in order to create a clear picture (29). Endogenous GCs for example can both promote and suppress T cell immunity. GCs are involved in selection of T cell receptor (TCR) self-affinities in the thymus, T cell trafficking, suppression of Th1 and Th17 responses, while promoting Th2 and Treg activity and memory T cell differentiation by means of target gene (e.g. cytokine) expression and transcription factor activation via the intracellular GC receptor (30–32). GCs also have the potential to inhibit T cell activation by interference with TCR signaling (29, 33, 34).

The most impressive changes in levels of T cell subpopulation in our study were decreased percentages of Th1 in AD patients and Tc1 cells in AD and CAH patients compared to those of controls. Moreover, we observed a downregulation of the percentage of IL-4^+^ Th2 and Tc2 cells as well as Th17 and Tc17 cells in patients with AD. Previously published analysis of chemokine profiles in small cohorts of up to 15 patients with autoimmune AD indicated a Th1 dominant chemokine profile, which we however could not confirm in our study (35, 36). Downregulation of Th1, Th2 and Th17 subsets however fits PAI patients’ susceptibility to infections as Th1 and Th2 cells play important roles in identification and eradication of intracellular pathogens such as parasites, viruses and bacteria and also the defensive mechanisms of Th17 cells particularly at the mucosal and epithelial barrier are not to be neglected (14, 37–39). Especially the lower expression of IFNγ, which is a key cytokine involved in several cellular programs, signaling mechanisms and surveillance of immune function, could have detrimental effects on immune protective mechanisms (40).

Our data additionally revealed a lower percentage of Tregs, especially in patients after BADx and those with AD. Defective suppressor function of Tregs has previously been shown in a cohort of patients with polyendocrine autoimmune syndrome and the critical role of Tregs in maintenance of self-tolerance and immune cell homeostasis is widely known (41, 42). Differences from percentage expression in healthy controls can therefore be at least partially explained by the pathophysiology of the disease in AD patients, however similar disparities in patients after BADx indicate other accountable factors. Previous studies demonstrated development of autoimmune disease in patients successfully treated for CS, which the authors hypothesized might have been masked in patients with glucocorticoid excess (25, 26). In this study, we could now observe a reduction in the percentage of Tregs in all PAI subgroups, which could provide an additional explanation for a higher prevalence of autoimmune diseases in these patient subgroups.

Further effects on immunophenotype discovered are an upregulation of the percentage of Th9 cells in the subgroup of patients with CAH and those after BADx compared to patients with AD and healthy controls, which might be relevant regarding patients’ T cell-dependent allergic and antiparasitic immune response (43). We could also observe an upregulation of the percentage of Th22 and Tc22 cells in patients after BADx compared to the other two subsets of PAI patients and healthy controls.

Moreover, we could demonstrate an inhibition of NK cell cytotoxicity to varying degrees in AD and after BADx but with only minimal changes in the subset of CAH patients (**Figure 2A**). NK cells comprise a crucial barrier of the innate immune system by their early production of cytokines and chemokines and cell lysis potential irrespective of prior sensitization (44). Previous publications have demonstrated that GCs influence NK cell function through epigenetic modifications resulting in dysregulation of effector protein expression and hence inhibition of NK cell cytotoxicity, at least partially explaining the effect of chronic stress on incidence of infections and cancer (44–46). GCs however can also – dependent on the cytokine profile present – support NK cell proliferation and reactivity (35). Our data supports this hypothesis. On the one hand, GC treatment in PAI patients seems to result in dysregulation of NK cell receptor (NKCR) expression, meaning an upregulation of inhibitory receptors such as CD94, while the percentage of activating receptor (e.g. NKG2D, NKp30, Nkp46) positive NK cells was reduced (**Figures 2D-I**). On the other hand, patients with PAI show an upregulation of CD3^-^CD56^+^ NK cells and an upregulation of the degranulation marker CD107a (**Figure 2C**). This data suggests a generally activated phenotype of NK cells in PAI patients with however a lack of actual cytotoxic function in *in vitro* testing. This might be interpreted as a failure to compensate the dysregulation of NKCR expression. Comparing the different subtypes of PAI, it becomes apparent, that most changes in NK cell levels, NKCR and degranulation marker expression as well as NKCC are present in the subgroup of BADx and AD patients, while CAH patients consistently present with only slight changes in NK cell phenotype.

It has previously been shown that in patients with CAH, hormone precursors accumulating upstream of the genetic defect in steroidogenesis are able to potently bind and transactivate the human GC receptor, explaining cortisol functions in patients even with null mutations (47). This could possibly explain why NKCC is less affected in this patient subgroup. In contrast to patients with AD and after BADx, CAH patients do not lack adrenal androgen production, but rather have elevated adrenal androgen concentrations potentially influencing their immunophenotype.

It is known that sex hormones are variables influencing the immune system as well as the susceptibility to all types of diseases and malignancies (48). Interestingly, however, we did not observe any gender differences in none of the PAI subtypes (data not shown). We did however previously demonstrate that adrenal-derived 11-oxygenated 19-carbon (11oxC19) steroids, which are known to be the dominant androgens in classic 21-hydroxylase deficiency, are also elevated in CAH patients with good therapeutic control (49, 50). As AKR1C3 is expressed on human PBMCs and 11oxC19 androgens the primary substrate of AKR1C3 expressed on human NK cells, differences in immunophenotype of CAH patients in comparison to those with AD or after BADx might also be conveyed by this subset of steroids, despite the sole effect of GC substitution therapy (51).

The pathophysiological differences and disparities in etiology of these different types of PAI promote the idea of further factors influencing the distinct differences in immunophenotype in the different PAI groups. In the subgroup of patients after BADx a long-term imprinting effect of the previous GC excess on the immune system might play a role. Patients with CS in remission show significant improvements in comorbidities in follow-ups, depending on the extent and possibly also the total duration of hypercortisolism (52). Nonetheless, despite long-term remission, comorbidities, risk of infectious complications and mortality remain higher compared to the general population (53, 54). Further influencing factors might also be the use and dose of mineralocorticoid replacement via action at the mineralocorticoid receptor (55, 56) or direct effects of ACTH as melanocortin receptors are also known to be expressed on most immune cell lineages (57).

Limitations of this study include variability in the timeframe of sampling including both the exact time of day (time frame from 8-12 am) as well as seasons (collected over the whole year), however this time-dependent bias is present in all groups and was shown to be able to be neglected in whole group comparisons. Handling, isolation of PBMCs and sample storage was standardized as well as lay and transport times and duration of sample processing.

To conclude, this manuscript presents novel data on immunophenotypic differences in subsets of PAI of differential etiology. Our data highlights the importance of further fundamental analysis of potential influencing factors of immune regulation, such as imprinting effects of long-term GC excess and effects of elevated GC and androgen precursors or 11oxC19 concentrations.

## Data availability statement

The raw data supporting the conclusions of this article will be made available by the authors, without undue reservation.

## Ethics statement

The studies involving humans were approved by Ethikkommission der Medizinischen Fakultät der LMU München. The studies were conducted in accordance with the local legislation and institutional requirements. The participants provided their written informed consent to participate in this study.

## Author contributions

HN: Conceptualization, Data curation, Formal Analysis, Funding acquisition, Investigation, Methodology, Visualization, Writing – original draft, Writing – review & editing. TMS: Conceptualization, Data curation, Formal Analysis, Investigation, Methodology, Visualization, Writing – original draft. JJ: Formal Analysis, Investigation, Writing – review & editing. AG: Methodology, Writing – review & editing. HS: Methodology, Writing – review & editing. SZ: Data curation, Writing – review & editing. FV: Methodology, Writing – review & editing. LT: Methodology, Writing – review & editing. MA: Methodology, Writing – review & editing. CL: Methodology, Writing – review & editing. SK: Methodology, Writing – review & editing. SR: Methodology, Writing – review & editing. FB: Methodology, Writing – review & editing. MR: Funding acquisition, Methodology, Project administration, Resources, Supervision, Writing – review & editing. LB: Funding acquisition, Methodology, Project administration, Resources, Supervision, Writing – original draft, Writing – review & editing. NR: Funding acquisition, Methodology, Project administration, Resources, Supervision, Writing – original draft, Writing – review & editing.

## Supplementary material

Supplementary Figure 1 Gating strategy for flow cytometric analysis of NK- and T cells. Gating strategy for both NK- and T cell subsets and NK cell surface receptors.

## Glossary

**Table:**

ACTH	adrenocorticotropic hormone
AD	Addison’s disease
ARNTL	aryl hydrocarbon receptor nuclear translocator-like protein 1
BADx	bilateral adrenalectomy
Bio AI/DSD	registry and biobank for adrenal insufficiency and differences of sex development
CAH	congenital adrenal hyperplasia
cDNA	complementary deoxyribonucleic acid
CLOCK	circadian locomotor output cycles kaput
CREB1	CAMP Responsive Element Binding Protein 1
CRY1/2	cryptochrome circadian regulator ½
CS	Cushing’s syndrome
CT	cycle threshold
DPBS	Dulbecco’s Balanced Salt Solution
DMSO	dimethyl sulfoxide
E	effector-cells
eGFP	enhanced green-fluorescent protein
FCS	fetal calf serum
GC	glucocorticoid
HC	hydrocortisone
IFNγ	Interferon-gamma
IL-4/9/17/22	Interleukin-4/9/17/22
IQR	interquartile range
NK	cell natural killer cell
NKCC	natural killer cell cytotoxicity
NKCR	NK cell receptor
NKG2D	natural killer group 2D
NKp30/46	NK cell-protein with 30/46 kilodalton
NR1D1	nuclear receptor subfamily 1 group D member 1
PAI	primary adrenal insufficiency
PBMC	peripheral blood mononuclear cell
PCR	polymerase chain reaction
PER3	Period Circadian Regulator 3
PFA	paraformaldehyde
PKC	protein kinase C
PMA	phorbol myristate acetate
Py	patient years
RNA	ribonucleic acid
T	targeT cells
Tc-cell	cytotoxic T cell
TCR	T cell receptor
Th-cell	T helper cell
TIMELESS	Timeless Circadian Regulator
WEE1	WEE1 G2 Checkpoint Kinase
