# Supplementary figures and images for: Major immunophenotypic abnormalities in patients with primary adrenal insufficiency of different etiology
Source: Front Immunol. 2023 Nov 15;14:1275828. doi: 10.3389/fimmu.2023.1275828 (PMC10690587; doi:10.3389/fimmu.2023.1275828)

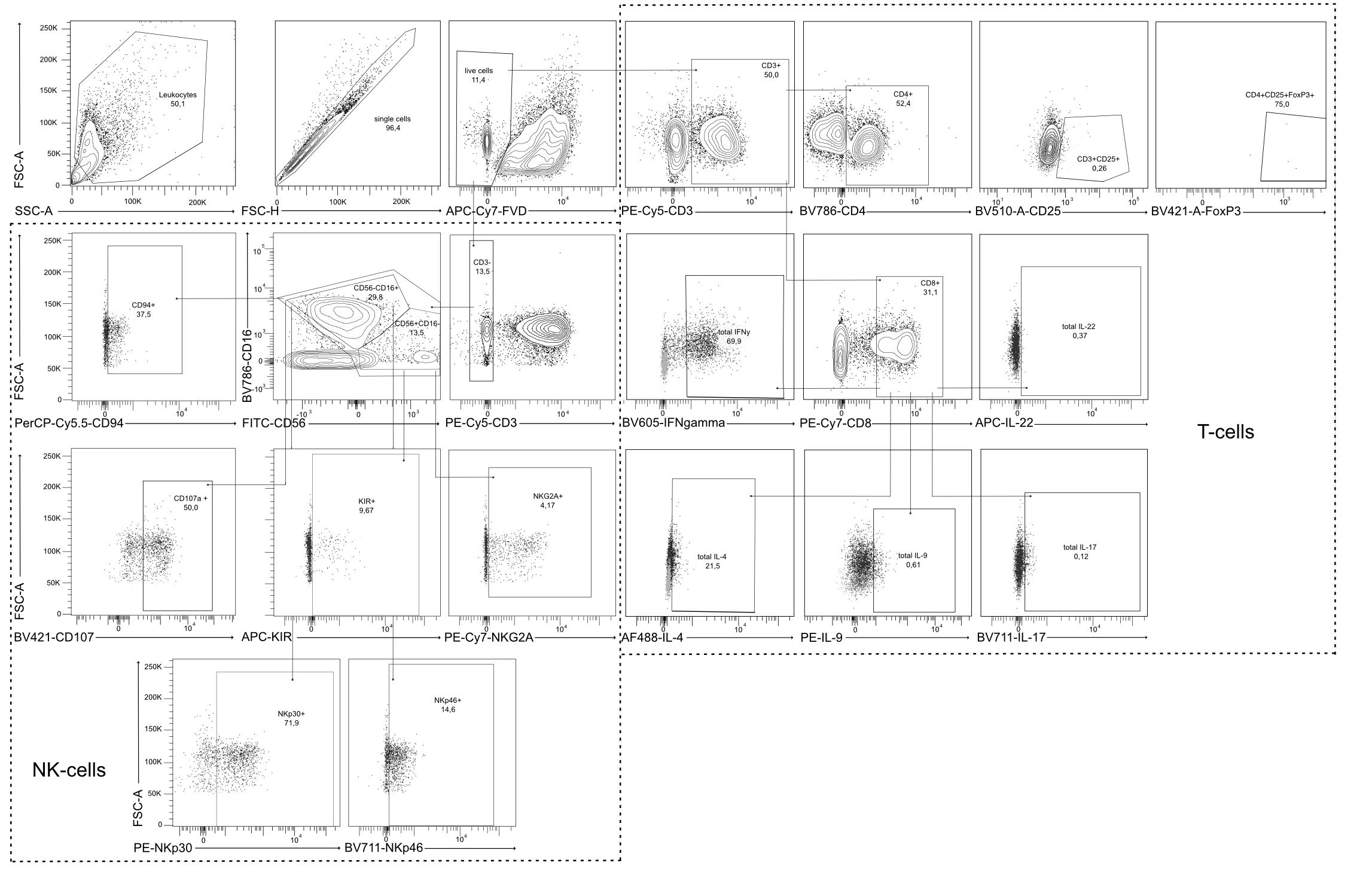

Supplement: Supplementary Figure 1 — Gating strategy for flow cytometric analysis of NK- and T cells. Gating strategy for both NK- and T cell subsets and NK cell surface receptors. [file Image_1.tiff]
