# Supplementary material for: Major immunophenotypic abnormalities in patients with primary adrenal insufficiency of different etiology
Source: Front Immunol. 2023 Nov 15;14:1275828. doi: 10.3389/fimmu.2023.1275828 (PMC10690587; doi:10.3389/fimmu.2023.1275828)
Supplement: Supplementary file 2 [file Table_1.docx]

**Supplemental Material**

**Table S1. Differences in T cell subsets in subgroups of PAI patients compared to healthy controls (including unstimulated conditions).**

| T-cells | CAH | BADx | AD | controls | p-value |
| --- | --- | --- | --- | --- | --- |
| CD3+ T-cells [U] | 63.0 (56.3 – 69.4) | 55.9 (46.2 – 63.9) | 59.5 (55.4 – 63.9) | 62.4 (57.9 – 67.7) | 0.0091 |
| CD3+ T-cells [S] | 61.5 (58.0 – 69.1) | 53.7 (47.5 – 61.0) | 57.3 (51.8 – 62.3) | 57.4 (52.5 -63.4) | 0.0063 |
| Δ CD3+ T-cells | -0.7 (-2.9 – 1.8) | -2.8 (-4.2 – (-0.6)) | -1.3 (-3.2 – 0.3) | -3.8 (-6.0 – (-2.1)) | <0.0001 |
| CD4+ T-cells [U] | 64.6 (57.3 – 74.6) | 68.9 (56.8 – 84.5) | 79.6 (75.3 – 85.0) | 73.9 (65.6 – 81.3) | 0.0011 |
| CD4+ T-cells [S] | 68.2 (60.7 – 75.9) | 63.9 (49.0 – 82.7) | 79.4 (73.5 – 84.5) | 77.1 (71.2 – 83.7) | 0.0003 |
| Δ CD4+ T-cells | 1.3 (-0.5 – 4.5) | 3.2 (1.0 – 5.8) | 0.2 (-1.4 – 2.5) | 4.0 (2.0 – 5.5) | 0.0002 |
| IFN-γ+ T_H_-cells [U] | 1.4 (0.6 – 3.6) | 8.6 (5.6 – 13.0) | 1.6 (0.8 – 3.8) | 8.8 (3.4 – 11.4) | <0.0001 |
| IFN-γ+ T_H_-cells [S] | 12.9 (7.7 – 19.0) | 17.4 (8.9 – 25.9) | 8.1 (4.8 – 16.4) | 18.0 (10.4 – 28.0) | 0.0026 |
| Δ IFN-γ+ T_H_-cells | 7.8 (5.4 – 17.3) | 6.1 (1.5 – 15.7) | 4.7 (2.2 – 14.4) | 8.9 (3.2 – 18.4) | 0.3857 |
| IL-17+ TH-cells [U] | 8.2 (3.4 – 15.5) | 7.3 (5.0 – 8.9) | 1.5 (0.7 – 2.2) | 10.2 (7.1 – 12.8) | <0.0001 |
| IL-17+ TH-cells [S] | 9.6 (5.1 – 15.6) | 11.4 (6.1 – 13.7) | 1.7 (1.2 – 2.4) | 8.7 (11.1 – 13.6) | <0.0001 |
| Δ IL-17+ TH-cells | 0.2 (-1.6 – 2.8) | 3.2 (1.7 – 6.0) | 0.6 (-0.1 – 0.9) | 1.4 (0.1 – 2.8) | 0.0006 |
| IL-4+ T_H_-cells [U] | 1.3 (0.5 – 2.3) | 3.5 (1.0 – 8.4) | 0.2 (0.1 – 0.5) | 1.6 (1.0 – 3.9) | <0.0001 |
| IL-4+ T_H_-cells [S] | 3.4 (2.4 – 5.3) | 5.8 (3.7 – 10.1) | 2.4 (1.6 – 3.9) | 4.4 (3.0 – 6.2) | 0.0011 |
| Δ IL-4+ T_H_-cells | 1.5 (0.9 – 3.1) | 1.8 (0.8 – 2.9) | 2.1 (1.4 – 3.4) | 2.1 (1.1 – 3.1) | 0.6471 |
| IL-9+ T_H_-cells [U] | 1.3 (0.7 – 3.4) | 1.8 (0.7 – 6.1) | 0.2 (0.1 – 0.4) | 0.3 (0.1 – 0.7) | <0.0001 |
| IL-9+ T_H_-cells [S] | 2.1 (0.9 – 3.8) | 3.3 (1.2 – 6.5) | 0.5 (0.2 – 0.7) | 0.2 (0.2 – 0.6) | <0.0001 |
| Δ IL-9+ T_H_-cells | 0.3 (-0.4 – 1.2) | 0.6 (-0.1 – 1.5) | 0.2 (0.01 – 0.4) | 0.04 (-0.1 – 0.2) | 0.0368 |
| IL-22+ T_H_-cells [U] | 0.3 (0.1 – 0.8) | 0.9 (0.4 – 2.2) | 0.2 (0.1 – 0.3) | 0.7 (0.5 – 0.9) | <0.0001 |
| IL-22+ T_H_-cells [S] | 0.6 (0.4 – 1.1) | 2.9 (0.9 – 5.4) | 0.4 (0.2 – 1.0) | 0.8 (0.5 – 1.2) | <0.0001 |
| Δ IL-22+ T_H_-cells | 0.3 (0.1 – 0.6) | 1.6 (0.5 – 3.0) | 0.3 (0.2 – 0.8) | 0.1 (-0.1 – 0.4) | <0.0001 |
| CD8+ T-cells [U] | 26.8 (16.1 – 30.5) | 16.2 (8.5 – 27.5) | 9.6 (5.7 – 12.8) | 20.0 (14.3 – 26.1) | <0.0001 |
| CD8+ T-cells [S] | 17.6 (12.4 – 23.4) | 22.5 (13.2 – 41.7) | 14.7 (9.7 – 19.0) | 7.8 (13.1 – 16.5) | 0.0009 |
| Δ CD8+ T-cells | .6.4 (-10.5 – (-4.8) | -6.2 (-10.6 – (-2.9) | -3.7 (-6.3 – (-2.3) | -6.9 (-8.8 – (-5.2) | 0.0142 |
| IFN-γ+ T_C_-cells [U] | 1.3 (0.3 – 3.8) | 9.3 (6.1 – 14.6) | 1.9 (0.8 – 5.2) | 11.2 (5.1 – 13.8) | <0.0001 |
| IFN-γ+ T_C_-cells [S] | 39.2 (25.7 – 49.9) | 58.8 (30.2 – 66.1) | 39.0 (26.3 – 46.5) | 36.7 (56.2 – 69.2) | 0.0004 |
| Δ IFN-γ+ T_C_-cells | 32.7 (23.8 – 47.5) | 46.4 (25.0 – 52.8) | 37.0 (24.2 – 43.2) | 45.5 (29.6 – 57.8) | 0.0584 |
| IL-17+ T_C_-cells [U] | 7.9 (3.1 – 11.2) | 6.5 (4.4 – 9.6) | 1.9 (1.0 – 3.6) | 11.2 (7.7 – 13.7) | <0.0001 |
| IL-17+ T_C_-cells [S] | 12.6 (6.1 – 16.3) | 12.9 (8.4 – 18.2) | 1.9 (1.1 – 3.6) | 16.6 (13.5 – 18.9) | <0.0001 |
| Δ IL-17+ T_C_-cells | 3.8 (0.8 – 5.9) | 5.0 (2.4 – 8.4) | 0.0 (-0.4 – 0.7) | 5.5 (3.5 – 7.0) | <0.0001 |
| IL-4+ T_C_-cells [U] | 4.5 (2.3 – 8.2) | 11.4 (4.7 – 29.7) | 1.7 (0.7 – 3.7) | 6.8 (4.0 – 12.3) | <0.0001 |
| IL-4+ T_C_-cells [S] | 4.2 (3.0 – 7.0) | 14.5 (5.4 – 23.5) | 2.5 (1.2 – 5.9) | 6.8 (3.9 – 10.8) | <0.0001 |
| Δ IL-4+ T_C_-cells | -0.4 (-2.1 – 1.1) | 0.3 (-1.1 – 3.0) | 0.7 (0.2 – 3.3) | -0.8 (-2.2 – 0.5) | 0.0037 |
| IL-9+ T_C_-cells [U] | 2.2 (1.4 – 4.4) | 2.4 (0.9 – 4.9) | 0.6 (0.3 – 1.1) | 5.7 (2.5 – 11.1) | <0.0001 |
| IL-9+ T_C_-cells [S] | 7.3 (4.1 – 15.6) | 13.2 (6.4 – 18.6) | 3.4 (2.4 – 7.9) | 4.2 (2.2 -8.9) | <0.0001 |
| Δ IL-9+ T_C_-cells | 4.6 (1-9 – 9.7) | 8.3 (5.2 – 14.3) | 2.9 (1.4 – 6.7) | -0.5 (-3.0 – 1.8) | <0.0001 |
| IL-22+ T_C_-cells [U] | 0.2 (0.05 – 0.5) | 0.5 (0.1 – 1.6) | 0.05 (0.03 – 0.2) | 0.5 (0.3 – 0.7) | <0.0001 |
| IL-22+ T_C_-cells [S] | 0.3 (0.1 – 0.9) | 1.4 (0.6 – 5.3) | 0.2 (0.04 – 1.0) | 0.5 (0.3 – 0.8) | <0.0001 |
| Δ IL-22+ T_C_-cells | 0.06 (-0.02 – 0.3) | 0.9 (0.3 – 3.0) | 0.1 (0.02 – 0.7) | -0.02 (-0.07 – 0.2) | <0.0001 |

*T-cell analysis in unstimulated [U] and stimulated [S] conditions with calculated difference (delta) between both conditions.T_H_-cells were defined as CD3+CD4+ and T_C_-cells as CD3+CD8+ T-cells. Tregs were defined as CD25+FOXP3+ T_H_-cells. Data is presented as percentage of live cells with median (IQR). Statistical analysis included Kruskal-Wallis.*

**Table S2. Differences in NK cell subsets in subgroups of PAI patients compared to healthy controls (including unstimulated conditions).**

| NK cells | CAH | BADx | AD | controls | p-value |
| --- | --- | --- | --- | --- | --- |
| **NK cells** [U] | 84.2 (80.5 – 86.5) | 89.7 (85.8 – 95.1) | 89.7 (87.1 – 92.6) | 85.4 (82.2 – 88.8) | <0.0001 |
| NK cells [S] | 84.9 (82.6 – 87.3) | 90.7 (84.7 – 94.2) | 88.4 (85.4 – 90.4) | 85.1 (82.8 – 87.6) | <0.0001 |
| Δ NK cells | 0.5 (-1.0 – 1.8) | - 0.3 (-1.1 – 0.8) | -1.0 (-2.4 – 0.7) | -0.9 (-2.2 – 1.7) | 0.0423 |
| **CD94+** NK-cells [U] | 39.7 (30.7 – 50.6) | 63.2 (51.2 – 69.3) | 50.8 (43.5 – 57.4) | 24.0 (18.9 – 29.8) | <0.0001 |
| CD94+ NK-cells [S] | 43.6 (35.4 – 49.1) | 66.4 (55.9 – 76.1) | 53.6 (42.9 – 61.9) | 34.6 (27.5 – 42.0) | <0.0001 |
| Δ CD94+ NK-cells | 3.1 (-3.5 – 7.6) | 0.04 (0.01 – 0.06) | 0.03 (0.00 – 0.05) | 0.1 (0.1 – 0.1) | <0.0001 |
| **CD107+** NK-cells [U] | 31.9 (27.0 – 36.4) | 36.8 (31.1 – 46.5) | 30.0 (22.3 – 41.7) | 27.8 (20.0 – 37.7) | 0.0012 |
| CD107+ NK-cells [S] | 46.2 (42.0 – 50.8) | 67.7 (58.6 – 77.2) | 55.7 (50.9 – 63.4) | 44.0 (29.0 – 50.9) | <0.0001 |
| Δ CD107+ NK-cells | 16.0 (7.0 – 21.3) | 0.3 (0.2 – 0.3) | 0.2 (0.1 – 0.3) | 0.1 (0.0 – 0.2) | <0.0001 |
| **KIR+** NK-cells [U] | 6.2 (3.6 – 10.1) | 10.2 (6.66 – 14.5) | 10.1 (5.82 – 16.2) | 13.3 (9.17 – 19.8) | <0.0001 |
| KIR+ NK-cells [S] | 8.4 (5.3 – 10.7) | 12.2 (9.5 – 15.6) | 13.2 (7.2 – 18.4) | 15.7 (10.7 – 22.4) | <0.0001 |
| Δ KIR+ NK-cells | 2.0 (0.9 – 2.5) | 0.02 (0.01 – 0.03) | 0.01 (0.00 – 0.03) | 0.02 (0.00 – 0.08) | 0.8917 |
| **NKG2A+** NK-cells [U] | 21.7 (18.4 – 30.5) | 22.8 (13.3 – 36.3) | 19.7 (16.1 – 30.4) | 29.3 (22.0 – 33.3) | 0.0175 |
| NKG2A+ NK-cells [S] | 19.7 (16.7 – 23.4) | 20.2 (13.7 – 35.6) | 17.8 (13.7 – 28.6) | 25.9 (22.1 – 33.8) | 0.0008 |
| Δ NKG2A+ NK-cells | - 2.7 (-6.1 – -1.1) | -0.01 (-0.05 – 0.01) | -0.01 (-0.03 – 0.01) | -0.01 (-0.1 – 0.0) | 0.1443 |
| **NKG2D+** NK-cells [U] | 38.6 (33.4 – 50.4) | 34.5 (21.4 – 43.6) | 28.2 (23.0 – 38.7) | 53.1 (21.7 – 65.4) | 0.0034 |
| NKG2D+ NK-cells [S] | 35.1 (27.5 – 42.0) | 22.6 (15.8 – 27.6) | 21.5 (17.2 – 23.5) | 34.6 (12.8 – 43.5) | <0.0001 |
| Δ NKG2D+ NK-cells | -7.2 (-10.6 – -2.4) | -0.1 (-0.2 – (-0.1)) | -0.1 (-0.1 – 0.0) | -0.1 (-0.2 – (-0.1)) | 0.0009 |
| **NKp30+** NK-cells [U] | 18.2 (14.1 – 26.4) | 31.8 (16.1 – 46.7) | 25.9 (16.8 – 36.1) | 34.6 (28.0 – 44.3) | <0.0001 |
| NKp30+ NK-cells [S] | 21.6 (14.9 – 28.5) | 32.1 (19.9 – 41.6) | 25.4 (21.6 – 40.4) | 33.4 (28.0 – 42.1) | 0.0001 |
| Δ NKp30+ NK-cells | 0.0 (-1.1 – 4.2) | 0.02 (-0.02 – 0.05) | 0.03 (-0.01 – 0.05) | -0.02 (-0.05 – 0.03) | 0.0208 |
| **NKp46+** NK-cells [U] | 96.2 (92.7 – 97.1) | 85.4 (73.8 – 90.1) | 77.6 (75.4 – 84.6) | 97.9 (64.6 – 98.6) | <0.0001 |
| NKp46+ NK-cells [S] | 91.1 (86.1 – 93.3) | 69.6 (53.4 – 74.1) | 59.8 (57.7 – 62.3) | 92.5 (43.3 – 95.2) | <0.0001 |
| Δ NKp46+ NK-cells | -0.05 (-0.10 – -0,03) | -0.17 (-0.24 – -0.15) | -0.19 (-0.22 – -0.15) | -0.06 (-0.13 – -0.03 | <0.0001 |
| NK-cell analysis in unstimulated [U] and stimulated [S] conditions with calculated difference (delta) between both conditions. Total NK-cell number is shown as percentage of all live cells. NK-cells expressing each cluster of differentiations are shown as percentage of all natural killer cells. Data is presented as median (IQR). | | | | | |
